# Single Cell Transcriptome Helps Better Understanding Crosstalk in Diabetic Kidney Disease

**DOI:** 10.3389/fmed.2021.657614

**Published:** 2021-08-17

**Authors:** Chunyang Du, Yunzhuo Ren, Guixin Li, Yan Yang, Zhe Yan, Fang Yao

**Affiliations:** ^1^Key Laboratory of Kidney Diseases of Hebei Province, Department of Pathology, Hebei Medical University, Shijiazhuang, China; ^2^Department of Burn, The Second Hospital of Hebei Medical University, Shijiazhuang, China; ^3^Department of Nephrology, The Second Hospital of Hebei Medical University, Shijiazhuang, China

**Keywords:** single-cell RNA sequencing, crosstalk, diabetic kidney disease, glomerulus, tubular epithelial cell

## Abstract

Years of research revealed that crosstalk extensively existed among kidney cells, cell factors and metabolites and played an important role in the development of diabetic kidney disease (DKD). In the last few years, single-cell RNA sequencing (scRNA-seq) technology provided new insight into cellular heterogeneity and genetic susceptibility regarding DKD at cell-specific level. The studies based on scRNA-seq enable a much deeper understanding of cell-specific processes such as interaction between cells. In this paper, we aim to review recent progress in single cell transcriptomic analyses of DKD, particularly highlighting on intra- or extra-glomerular cell crosstalk, cellular targets and potential therapeutic strategies for DKD.

## Introduction

Diabetic Kidney Disease (DKD) is a microvascular complication associated with type I or type II diabetes. It has become a public issue and seriously threaten human health and lives. As the leading single cause of end-stage renal disease (ESRD) in many countries, such as the United States, DKD accounts for more than half of all patients enrolled in renal replacement therapy (RRT) programs (1). Although there has been a decline in the incidence of DKD over the past 30 years due to improved diabetes managements, the absolute risk of renal and cardiovascular morbidity and mortality remains overwhelmingly high (2–6). A deeper insight into the pathogenesis of DKD is required for innovative treatment strategies to prevent, arrest, and reverse DKD. Hyperglycemia is thought to be a major factor for diabetic complications and causes accumulation of toxic glucose derivatives (7, 8). However, hyperglycemia alone is not sufficient to the development of DKD since about only 30% of patients with type 1 diabetes mellitus (DM1) and 40% of patients with type 2 diabetes mellitus (DM2) develop this microvascular complication (1, 9). Family aggregation of DKD shown by independent familial studies in different populations suggests a genetic predisposition to DKD (10, 11). Moreover, patients with DKD are not always present with micro/macro-albuminuria. A large proportion of diabetic patients have declined renal function in absence of substantial proteinuria (12). The DKD heterogeneity suggested by the aforementioned evidences implies variant modulation of kidney function in diabetes and highlights the need for better biomarkers to predict the progressive kidney failure in the patients without heavy proteinuria.

Kidney is a highly complex organ consisting of about a million nephrons in humans which is composed of more than 40 different cell types (13, 14). The need for better understanding of the complex cell-to-cell interaction within or even beyond the heterogeneous kidney milieu comes naturally and rationally to reveal the complex mechanism underlying kidney organization, function and disease. The current clinical diagnoses for renal diseases as well as experimental researches on kidney depend largely on morphological cell identification and their known biomarkers. However, some important disease discriminative and prognostic features may not be effectively captured due to the highly operator-dependent microscopical observation and the limited biomarkers available. A single cell transcriptional profiling by a new set of technologies–single cell RNA-sequencing (scRNA-seq) has emerged in the last 10 years as a powerful approach helping to decipher complex information in cells and organs (15, 16). Here we aim to review cell-to-cell cross talk in DKD, particularly highlighting the latest insight gained by scRNA-seq researches.

## Brife Introduction of Single Cell RNA Sequencing

**Figure 1:**
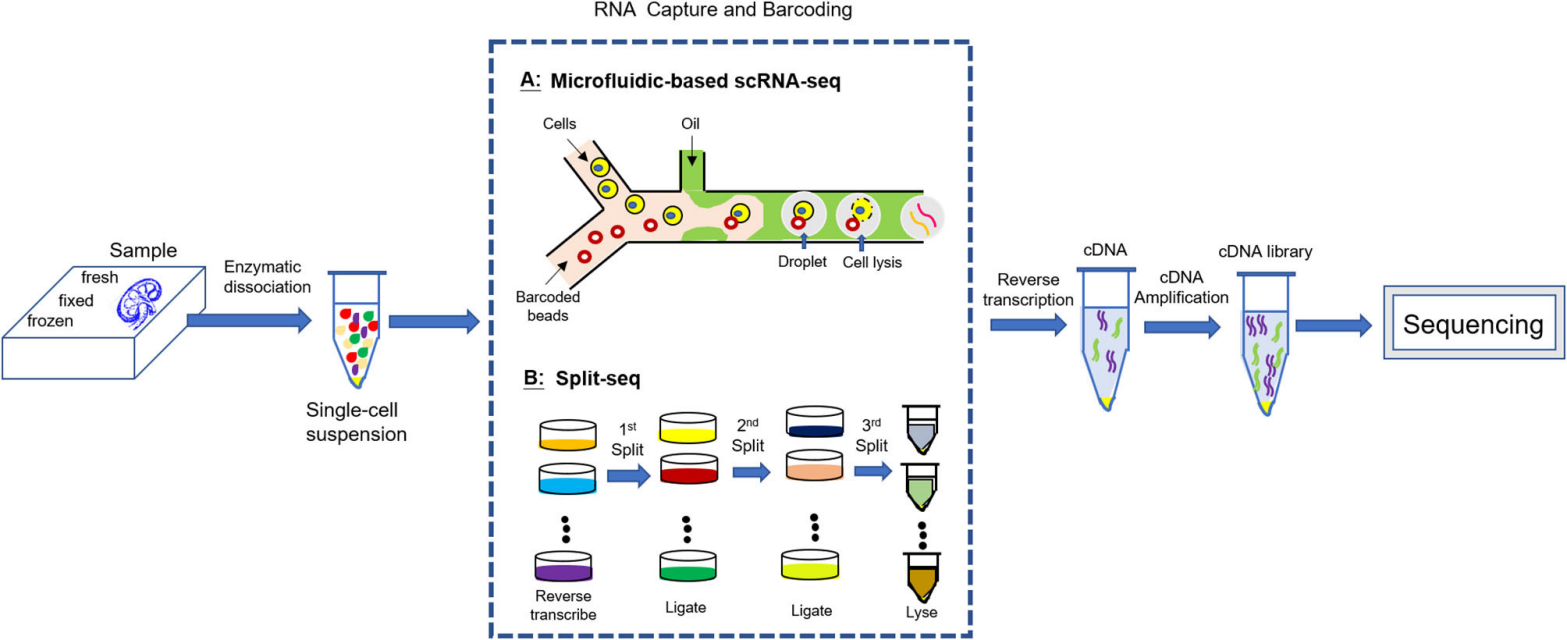
scRNA-seq experimental workflow. All scRNA-seq techniques include several common steps: enzymatic dissociation of the sample into a single cell suspension; individualized RNA capture (individual transcriptome barcoding); reverse transcription and transcriptome amplification; cDNA library preparation; sequencing.

scRNA-seq is a new set of technologies for genome wide RNA profiling of individual cells based on whole-genome-amplification (WGA) methods and next-generation sequencing (NGS) technologies (17–20). Before the invention of scRNA-seq, the genome-wide transcriptomic information primarily came from “bulk” RNA-seq, whose data represent an average of gene expression across individual cells and thus may mask some transcriptional information from less representative subpopulation. Compared with bulk RNA-seq, scRNA-seq provides more unbiased gene expression profiles at a single-cell resolution. The scRNA-seq methods have gained considerable progress over the last decade while the single cell DNA sequencing (scDNA-seq) has proven to be more challenging than RNA due to the fact that a single cell contains only two copies of each DNA molecule, but thousands of copies of most RNA molecules, which result in more technical error in scDNA-seq (15). All scRNA-seq techniques include several common steps: single cell isolation, cell lysis and RNA capture, reverse transcription and transcriptome amplification, cDNA library preparation, and sequencing and quantification. The most challenge for scRNA-seq is cell isolation and individualized RNA capture (individual transcriptome barcoding). Two barcoding strategies are suggested, either (1) the addition of a cell-specific barcode to each transcriptome following cell isolation (Microfluidics-based scRNA-seq), or alternatively (2) the addition of a unique index combination to each cell transcriptome without physical partitioning (e.g., Split-seq) (21–25) (Figure 1). Bio information from sequencing is intensively analyzed by computer and the final result is generation of a digital expression matrix including all detected gene expression in each individual cell. High throughput scRNA-seq data are processed to cluster cells and visualized by dimensionality reduction graph. Cell types are identified by examining known marker gene expression in each cluster and shown by the heatmap. Gene-gene correlation analysis helps to clarify the relationship between two marker genes within a cluster as well as the relationship of two marker genes from different clusters. Dynamic gene expression in single cell can be tracked along pseudotemporal trajectory corresponding to a biological process (e.g., development, differentiation, and disease progression). Key regulators for the dynamic gene expression can also be revealed by regulatory network analysis on transcription factors (26).

## Applications of Single Cell RNA Sequencing on Kidney Diseases

The knowledge regarding the transcriptional landscape of kidney in last 20 years was achieved largely from the “bulk” RNA-seq, which, though highly informative, is limited to describing an average transcriptome across a cell population in a bulk renal tissue or even in finely separated kidney compartments and thus masks or skews signals of interest (26–29). The comprehensive definition of cell types and states cooperating with examination of gene expression in specific cells by scRNA-seq makes it possible for determining specific disease-causal cells and genes. Park et al. performed scRNA-seq on kidneys from healthy male mice and unexpectedly identified a transitional cell type between intercalated cells (ICs) and principal cells (PCs) in collecting duct (30). They further demonstrated this IC-to-PC transition is mediated by Notch signaling and the shift toward the PC fate is the likely cause of metabolic acidosis in chronic kidney disease (CKD) (30). Recently Liao et al. delineated a transcriptomic map of human kidney cells basing on scRNA-seq analysis of normal human kidney (31). Another single-cell transcriptome profiling performed on human kidney allograft biopsy specimens (32) helped mapping previously defined rejection-associated genes to single cell types and revealed paracrine signaling pathway between infiltrating leukocytes and kidney parenchyma (33). A more recent scRNA-seq performed on purified glomeruli from four common kidney injury models (nephrotoxic serum nephritis, diabetes, doxorubicin toxicity and CD2AP deficiency) generated comprehensive snapshots of the altered genetic landscapes in multiple models (34). This research provided new insights into kidney injuries, such as that mesangial cell may shape the characters of the inflammation and wound healing programs in response to distinct types of injuries; persistent mesangial reaction may drive the chronic decline of kidney function in many disease; Hippo pathway is critical for podocyte repair in kidney injuries (34). In a scRNA-seq research on isolated glomerular cells from experimental diabetic mice, the unique trajectory analysis of scRNA-seq revealed dynamic changes of gene expression in endothelial and mesangial cells in diabetic mice (35). Subramanian et al. in a research regarding kidney organoids presented a comprehensive census of human organoids enabled by scRNA-seq in comparison to human adult and fetal kidneys (36). This census achieved some quantitative insight into organoid reproducibility and the data validated the faithfulness of kidney organoids from four different patient-derived induced pluripotent stem cell (iPSC) lines (AS, N1, N2, and ThF), which serve as surrogates of human kidney tissue for the study of a broad array of kidney diseases. The census data also addressed an issue of organoid quality, suggesting that the elimination of off-target cells may also benefit organoid maturity (36). In renal tumor research field, Young et al. studied Wilms' tumor, clear cell, and papillary renal cell carcinoma in relation to healthy fetal, pediatric, adolescent, and adult kidneys, as well as ureters (37). By analyzing tumor composition with scRNA-seq, they verified the hypothesis that Wilms' tumor cells are aberrant fetal cells and defined cancer-associated normal cells as well as delineated a complex VEGF signaling circuit (38). The power of scRNA-seq is not just to identify or catalog cells. It can help predict treatment outcomes and guide therapy. In a scRNA-seq research carried out by Park group, intratumoral heterogeneity was examined between a pair of primary renal cell carcinoma and its lung metastasis (37). The activation of drug target pathways demonstrated considerable variability between the primary and metastatic sites, as well as among individual cancer cells within each site. Guided by scRNA-seq analysis, a combinatorial regimen co-targeting two mutually exclusive pathways for the metastatic cancer cells gained better treatment efficacy over monotherapy (37).

More recently, Humphreys group (39) and Susztak group (40) both profiled kidney transcriptome and chromatin accessibility with sc/nRNA-seq and single nucleus assay for transposase-accessible chromatin using sequencing (snATAC-seq) respectively in their researches. These two multi-omics researches revealed the powerful potential of joint profiling with scRNA-seq in understanding kidney disease and development.

## Challenges to Single Cell RNA Sequencing on Kidney Research

Despite the tremendous development of technology, scRNA-seq research is still facing many challenges. Cell isolation and individualized RNA capture remain to be the most challenges, since enzymatic dissociation protocols usually compromise cell viability and adult kidneys are relatively dense with matrix, thus the quality of single cell suspension does not accurately reflect the transcriptional state of each cell before dissociation (26). The possibility of selective cell loss during tissue dissociation and the transcriptional stress response induced by the proteolytic process as well as RNA degradation lead to bias. This may partly account for the failure in detecting about 25% of single kidney cells in sequencing in the work by Park et al. (30). The failure in detecting podocytes in transplant biopsy might be explained by the similar reason (33). The dissociation protocols need to be optimized responding to different kidney origins, since in some diseases, the injured podocytes are more susceptible to loss during enzymatic digestion whereas mesangial cells are less effectively isolated and captured due to the increased matrix. Cold dissociation was recommended by researchers as digestion on ice avoided stress and achieved more abundant cell types than warm dissociation at 37°C (41). The strategy adopted by Chen et al. and Karaiskos et al. in their experiments may partly correct some dissociative artifacts by dissecting specific portion of kidney tissues (e.g., proximal tubules or glomeruli) (42, 43). Human biopsy from patient is another challenge to scRNA-seq since scRNA-seq requires a relatively large number of cells for the automatic cell separation and capture system.

Recently, single nuclear sequencing (snRNASeq) rise popular as an alternative to scRNA-seq for its obvious advantages in gaining good quality nuclei from snap frozen sample while bypassing the proteolytic process at 37°C (44–46). But there were researches implied T, B, and NK lymphocytes were underrepresented in the single-nucleus libraries (40, 42, 44). When interpreting the results of scRNA-seq, protocol-specific biases must be taken into consideration as cryopreservation of dissociated cells results in a major loss of epithelial cell types while methanol fixation maintains the cellular composition but suffers from ambient RNA leakage (41).

In addition to the crucial step of cell dissociation, a successful scRNA-seq is also challenged through the computational workflow. Depending on the platform of choice, researchers individualize their own procedural steps and choose specific analytic tools for data processing from the step of raw counts normalization to feature selection, dimensionality reduction, and clustering. When inferring cell-cell communication from transcriptomics, most of the researchers built the lists of ligand-receptor pairs from multiple databases and literature curation. Armingol et al. collated publicly available lists into a single ligand-receptor pair repository to facilitate further use and comparison (47). However, integrating multiple sources of data is challenging and requires reconciliation of the different ways ligand–receptor pair confidence was assessed or how orthologs were determined (47). To identify the genes associated with cell communication, the gene expression matrix generated by scRNA-seq is filtered by ligand-receptor pairs and a communication score for each ligand-receptor pair is computed using their gene expression levels [function f(L, R), where L and R are the expression values of the ligand and the receptor, respectively] (47). An aggregated communication score is also computed to estimate the overall state of interaction between respective samples or cells and the final results are visualized as Circos plot, network, Heatmap, etc. (47). There may exist some false positives or negatives in the inferred cell communication due to the data-driven inference process, which can lead to different results depending on the strategy adopted (47). Although some powerful computational tools such as CellChat, CellPhoneDB, NicheNet help to minimize the false discoveries (48–50), the findings derived from scRNA-seq need to be validated by experimental tests including immunohistochemistry, western blot and other functional assessment.

## Intraglomerular Crosstalk in DKD

**Figure 2:**
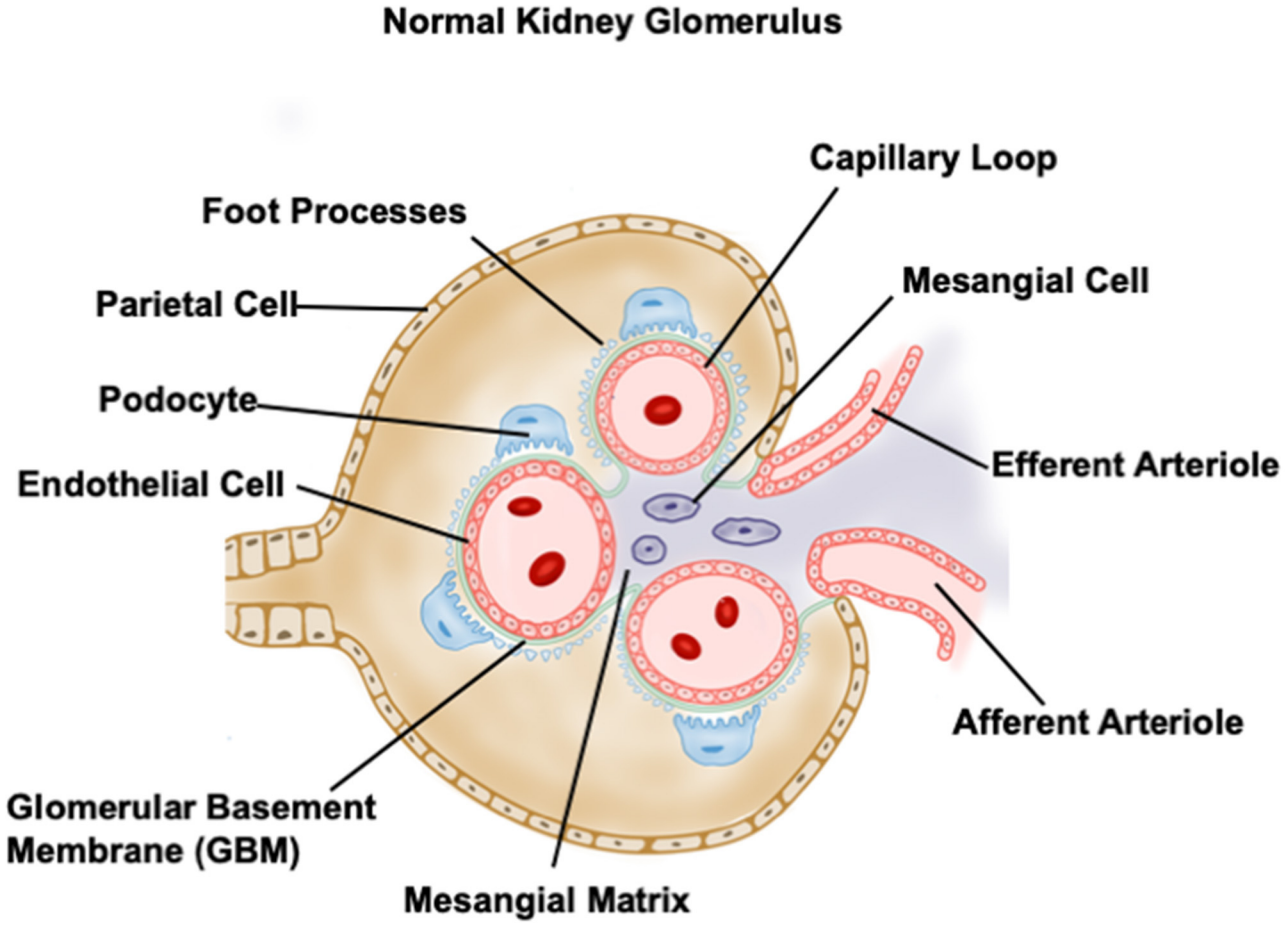
Diagrammed normal glomerular structure.

Glomerulus is a highly organized complex with two major compartments, the glomerular capillary tuft and the so-called Bowman's capsule which surrounds the capillary tuft. Podocytes wrap around the glomerular capillary with foot processes, which are connected by slit diaphragms bridging the filtration slits. The intraglomerular mesangial cells reside between capillary loops in close contact with glomerular endothelium. Parietal epithelial cells (PECs) compose the outer layer of the capsule, directly connecting to proximal tubules. The formation and maintenance of the glomerular filtration barrier require the communication within glomerulus including a multidirectional crosstalk between podocytes, mesangial cells and endothelial cells as well as PECs (51–57). The normal glomerular structure is shown in Figure 2 (9).

In DKD, the glomeruli are exposed to various noxious stimuli such as high glucose, fatty acids, uric acid, growth factors, cytokines, and hormones, which dysregulate the communication in glomerulus.

### Crosstalk Between Podocyte and Glomerular Endothelial Cell (GEC)

Studies have shown that the severity of DKD is correlated with endothelial dysfunction in T1DM and T2DM (58, 59). The growth and survival of GECs are regulated by paracrine vascular endothelial growth factor alpha signaling (VEGFA-VEGFR) (60, 61). Podocyte VEGFA deletion results in the development of heavy proteinuria, marked glomerulosclerosis and glomerular cell apoptosis (62). Conversely, increased podocyte-derived VEGFA was shown to be deleterious in non-diabetic mice, and the injury was further exacerbated with diabetes induction, resulting in advanced glomerulopathy with massive proteinuria (63). Podocyte-produced angiopoietins, functioning as endothelial cell-protective factors in diabetes, mediate podocyte-to-endothelial crosstalk and are critical for modulating the vascular response after the onset of diabetes (61, 64). Angiopoietin-1 (Angpt1) is expressed by podocytes and mesangial cells and its cognate tyrosine kinase receptor, Tie2/Tek is expressed by the glomerular endothelial cells. Angpt1 and Angpt2 both bind to Tie2 receptor and have classically been viewed as having opposing effects on microvascular development, with Angpt1 stabilizing the vasculature, and Angpt2 antagonizing these effects by binding to the Tie2 receptor in a competitive manner (52). Endothelin-1 (Edn-1)/endothelin receptor type A (EdnRA) axis has been demonstrated to be a causative regulator in promoting endothelial cell dysfunction in human biopsies and experimental model of FSGS, and is responsible for the loss of glomerular endothelial glycocalyx by increased degradation of glycosaminoglycans (65–67). In diabetes, EdnRA in GECs is activated by increased circulating Edn-1 or local Edn-1, resulting in mitochondrial stress and endothelial dysfunction (68, 69).

A similar stressed endothelial-to-podocyte crosstalk *via* mitochondrial oxidative stress in endothelial cells downstream from Edn-1/EdnRA could also underlie segmental lesions in DKD and highlighted a potential mechanism for the proven renoprotective activities of EdnRA inhibitors (70, 71). Activated protein C (APC) formation, which is regulated by endothelial thrombomodulin, is required for podocyte viability (72). Loss of thrombomodulin-dependent APC formation interrupts crosstalk between the vascular compartment and podocytes, causing glomerular apoptosis and diabetic nephropathy (72). Krüppel-like factor 2 (KLF2) is a shear stress-inducible transcription factor and has endoprotective effects on podocytes. Endothelial cell knockout of KLF2 resulted in reduction of the endothelial glycocalyx and podocyte injury in diabetes (73). Recently, a study showed that endothelial derived exosomes, which are enriched with TGF-β1 mRNA, could mediate epithelial-to-mesenchymal transition (EMT) and induce dysfunction of podocytes in a paracrine manner with activation of canonical Wnt/β-catenin signaling (74).

### Crosstalk Between Podocyte and Parietal Epithelial Cell (PECs)

Enlightened by delicate balance between visceral and parietal epithelial cells across Bowman's space, the crosstalk between podocytes and PECs are thought to be as equally important as the tight interaction between endothelial cells and podocytes across the glomerular basement membrane (GBM) (75). Indeed, multiple studies have consistently corroborated that the depleted podocytes can be regenerated *via* differentiation of the adjacent PECs, which serve as the local progenitors of podocytes to reconstitute the podocyte population upon glomerular injury and podocyte loss (53, 76–83). Injured podocytes secrete heparin-binding epidermal growth factor-like growth factor (HB-EGF), which in turn stimulates and promotes the proliferation of PECs while disturbs their compensatory differentiation toward podocytes (77, 83). Another growth factor, insulin-like growth factor-1(IGF-1) has been proved more critical for promoting the differentiation of PECs into podocytes (83). A 3D multiscale modeling study suggested that promoting PECs differentiation are as equivalently important as amelioration of glomerulus stress for podocyte regeneration (83).

### Crosstalk Between Glomerular Mesangial Cell (GMC) and Other Glomerular Cell Types

Mesangial cells are considered to be specialized pericytes due to their spatial intimacy with endothelial cells, thus functioning in stabilizing vasculature, synthesizing components of the basement membrane, and participating in controlling capillary vascular tone (84). The tight link between the fates of mesangial cell and endothelial cell has been well established by demonstrating the importance of platelet-derived growth factor B (PDGF-B) and its receptor PDGFR-β in the interaction of endothelial and mesangial cells (84–87). Recent evidences have revealed a significant role of exosomes as the messenger cargos for intercellular communications within glomerulus in DKD (88). Wu et al. demonstrated exosomes released by high glucose-treated GECs could promote α-smooth muscle actin (α-SMA) expression, proliferation and extracellular matrix protein overproduction in GMCs through the TGF-β1/Smad3 signaling pathway (89). In respect of the crosstalk between podocyte and mesangial cell, several signaling pathways have been suggested to be involved including VEGF, Edn-1, CCR7, and its ligand SLC/CCL21, PDGF, connective tissue growth factor (CTGF), hepatocyte growth factor (HGF) and TGF-β (84, 90). Among these signalings, VEGFA and nitric oxide (NO) are considered to play a pivotal role in driving the development of typical DKD lesions, causing as important effects on GMCs as those on endothelial cells. (91). The diabetic podocyte produces excessive VEGF in the setting of low endothelial NO and stimulates growth and proliferation of mesangial and endothelial cells, leading to increased extracellular matrix accumulation, hyperfiltration, and proteinuria (92). A recent research has shown that an intraglomerular crosstalk between mesangial cells and podocytes can inhibit physiological endoplasmic reticulum stress-associated degradation (ERAD) and suppress the phosphorylation of nephrin in podocytes, which thereby lead to podocyte injury under diabetic conditions (92).

The intraglomerular crosstalks are summarized in Table 1.

**Table 1:** Summary of the mediators for intraglomerular crosstalk in DKD.

**Crosstalk**	**Ligand/Receptor**	**Extracellular vesicles**	**Signal pathway**	**Pathological role in DKD**	**Reference**
Podocyte-GEC	VEGFA–VEGFR2			The expression of VEGFA and VEGFR2 is increased in early DKD, but with the loss of podocytes at later stage of DKD, the expression of VEGFA is also significantly decreased. The VEGFA-VEGFR2 signaling contribute to vascular rarefication and renal fibrosis in the development of DKD.	(3, 60–63, 91)
	Angpt1/2–Tie2			Decreased ratio of Angpt-1/Angpt-2 contributes to the development of DKD. Angpt-1 could retard the development of albuminuria as well as glomerular endothelial cell proliferation, whereas Angpt-2 has the opposite effects in DKD.	(3, 64)
	Edn-1–EdnRA			The expression of Edn-1 is upregulated in DKD and combined with the receptor EdnRA, which contributes to the mitochondrial dysfunction of endothelial cell and podocyte apoptosis.	(64, 66–69)
	SDF-1–CXCR4			SDF-1/CXCR4 axis is involved in the pathogenesis of glomerulosclerosis in case of type 2 diabetes. Inhibition of SDF-1significantly reduced diffuse glomerulosclerosis and prevented albuminuria in the diabetic model.	(70)
			ANGPTL4	An upregulation of podocyte secreted Angptl4 has described in experimental diabetic animal, which contributed to proteinuria and endothelial injury.	(3)
GEC-Podocyte	APC–PAR1/ EPCR/S1PR1			A loss of thrombomodulin-dependent protein C activation and aggravated glomerular apoptosis is described in diabetic mice. Increased levels of APC formation prevent podocyte apoptosis and downregulates coagulation and inflammation in DKD.	(72)
			KLF2	The expression of KLF2 is reduced in diabetic kidneys and it lack aggravates endothelial and podocyte injury in diabetic nephropathy.	(73)
			eNOS	A tight relation has been found between eNOS deficiency and a podocyte-specific injury and heavy albuminuria in advanced DKD.	(75)
			Endothelial glycocalyx	The damage of endothelial glycocalyx and shear-stress is observed in early DKD, and this damage altered organization of extracellular matrix.	(67)
		TGF-β1		The increased secretion of exosomes enriched with TGF-β1 mRNA probably mediates the EMT and dysfunction of podocytes through the Wnt/β-catenin signaling pathway.	(74)
Podocyte-PEC	HB-EGF–c-MET			Injured podocytes secrete HB-EGF, which in turn stimulates and promotes the proliferation of PECs while disturbs their compensatory differentiation toward podocytes.	(77, 82)
	IGF–IGFBPs			Dysregulation of the growth hormone/IGF system is found in early DKD and is associated with both glomerular hypertrophy and microalbuminuria.	(82)
GEC-GMC	PDGFB–PDGFR-β			PDGFR-β signaling is activated in glomeruli and tubule of diabetic mice.It may contribute to the progress of diabetic nephropathy, with an increase in oxidative stress and mesangial expansion.	(86, 87)
		TGF-β1		The increased secretion of exosomes enriched with TGF-β1 mRNA can promote α-SMA expression, proliferation and extracellular matrix protein overproduction in GMCs through the TGFβ1/Smad3 signaling pathway.	(88)
GMC-GEC			Integrin αvβ8	The integrin is expressed by mesangial cells, where it sequesters TGF-β, thereby reducing TGF-β signaling. Integrin αvβ8 and its ligand latent TGF-β protect kidney from glomerular dysfunction, endothelial apoptosis, and development of proteinuria, but the role of Integrin αvβ8 in DKD is unknown.	(91)
Podocyte-GMC			VEGF	The diabetic podocyte produces excessive VEGF in the setting of low endothelial NO and stimulates growth and proliferation of mesangial and endothelial cells, leading to increased extracellular matrix accumulation, hyperfiltration, and proteinuria.	(83)
GMC-Podocyte		TGF-β1		Exosomes derived from high-glucose-induced mesangial cells induced podocyte injury through the increased secretion of TGF-β and TGF-β1/PI3K-Akt signaling.	(83, 89)
			ERAD	ERAD-associated genes are downregulated in diabetic Glomeruli, and inhibition of ERAD processes could leading to the suppression of nephrin phosphorylation and podocytes injury under diabetic conditions.	(92)

*DKD, diabetic kidney disease; VEGF, vascular endothelial growth factor; VEGFR2, vascular endothelial growth factor receptor 2; Angpt1/2, angiopoietin-1/2; Tie2, angiopoietin 1 receptor; Edn-1, Endothelin-1; EdnRA, Endothelin receptor A; SDF-1, stromal cell-derived factor 1; CXCR4, C-X-C Chemokine Receptor Type 4; ANGPTL4, angiopoietin-like 4; IGF, insulin-like growth factor; IGFBP, insulin-like growth factor binding protein; HB-EGF, heparin-binding epidermal growth factor-like growth factor; c-MET, mesenchymal epithelial transition factor; APC, Activated protein C; PAR1, Protease-activated receptor 1; Sirt1, Sirtuin 1; EPCR, endothelial protein C receptor; S1PR1, Sphingosine 1-phosphate receptor 1; TGF-β1, Transforming growth factor β1; KLF2, Krüppel-like factor 2; eNOS, endothelial nitric oxide synthase; PDGFB, Platelet-derived growth factor B; PDGFRβ, platelet-derived growth factor receptor beta; IL-1β, Interleukin 1β; Integrin αvβ8, Integrin alphavbeta8; CCN1, Cellular communication network factor1; INSR, insulin receptors; ERAD, ER-associated protein degradation; NMN, nicotinamide mononucleotide; CCL2, C-C motif chemokine ligand 2; EMT, epithelial-mesenchymal transition; α-SMA, α-smooth muscle actin; GMC, glomerular mesangial cell; GEC, glomerular endothelial cell; PEC, parietal epithelial cell*.

### Findings Based on scRNA-Seq

By comparing the differential gene expression detected by scRNA-seq between specific cell types with the existing ligand-receptor database (http://fantom.gsc.riken.jp/5/) or the potential paracrine secreted ligand-to-membrane receptor pair list obtained by using Human Protein Atlas (https://www.proteinatlas.org/humanproteome/ secretome) and BIOGRID v3.5.165 (https://thebiogrid.org), the researchers can identify cell-cell crosstalk between glomerular cell types. To date, crosstalk data from scRNA-seq research regarding DKD are quite limited. Fu et al. performed scRNA-seq analysis on isolated glomerular cells from induced diabetic eNOS^−/−^ mice (35). They analyzed a total of 644 cells (326 control and 318 diabetic) with a median of 3,457 genes per cell (3,417 control and 3,509 diabetic). With less cells capture but much greater sequencing depth per cell (five-fold) in a plate-based platform, as the researchers mentioned, compared with the microdroplet-based platform, several ligand-receptor pairs in the glomerular cell were identified, some of which are well established (e.g., podocyte VEGFA-endothelial Flt1 and Kdr) while the others are less well characterized in the glomerular homeostasis (e.g., mesangial Epha3-endothelial Efna1) (35). The ligand-receptor pair analysis in scRNA-seq is unprecedentedly informative to suggest almost all the potential direct cell-to-cell crosstalks. Taking Fu et al.'s research for example, this analysis not only identified the established crosstalks such as VEGFA pair, which can be subjected to cross validation with the existing literatures, but also guide future exploration for those less established interaction, such as podocyte BMP7-mesangial BMPR2 pair and mesangial Angpt4-endothelial Tie1 pair, which are implied by insufficient literature, yet to be validated (35). Unlike the above-mentioned crosstalk of podocyte VEGFA-endothelial Flt1 and Kdr having handful supportive evidences, the literatures in respect of the cellular crosstalk involving bone morphogenetic protein-7 (BMP-7) in DKD are limited. BMP-7 in podocytes was reported possibly having protective effects against renal damage produced by hyperglycemia *via* the interaction with receptor BMPR2 (93, 94). But its cellular crosstalk in DKD *via* the interaction with its receptor needs more solid evidences. So does the pair of mesangial Angpt4- endothelial Tie1. Moreover, by comparing the diabetic ligand-receptor pairs with control, the changed crosstalk can be revealed, for example, the pairs of mesangial Angpt4-endothelia receptors were not detected in control, but showed in diabetes, even more prominent than other Angpt pairs (35).

In another scRNA-seq on cryopreserved human diabetic kidney samples, 23,980 single-nucleus transcriptomes were generated from three control and three early diabetic nephropathy samples (91). The researchers examined differentially expressed ligand–receptor pairs in glomerular cell types and found human diabetic mesangial cells had increased expression of CCN1 and SLIT3 (95). CCN1 responding extracellular protein, ITGAV, ITGB3, ITGB5 were expressed by podocytes, which interact with CCN1 and subsequently regulate tissue repair. Another CCN1 responding protein, ITGB3 was expressed by endothelial cells. ROBO2 was expressed by podocytes and endothelial cells, which interacted with SLIT3 to modulate cell migration. Diabetic endothelial cells also expressed increased LTBP1, which regulated targeting of latent TGF-β complexes (95). A scRNA-seq on mouse GMCs revealed GMCs having a high enrichment of genes involved in endothelial activity, supporting the long-existing notion that mesangial cells are specialized pericytes (96). Interestingly, the researchers also found that some mesangial cells express podocyte marker genes (e.g., Wt1) as well as endothelial cell marker genes (Tie2, Flk1, Flt1/ Vegfr1) (96).

When interpreting the data of ligand-receptor pair analysis, bias coming from misrepresented cell population must be taken into consideration. Podocytes, especially the injured podocytes are susceptible to loss during the dissociation process, which leads to limited podocytes detected, therefore results in the podocyte population underrepresented in most of the scRNA-seq research. It is also difficult to clearly identify mesangial cells due to the similarity between mesangial cells and stromal mesenchymal cells. Chung et al. found that several genes which were used in their study to identify mesangial cells are not specific while in some cases are specific to smooth muscle cells (SMCs) (Myh11, Rergl, Pln, and Olfr558) (34). They pointed that the study by Fu et al. was limited by the small number of cells, as the reason they referred were that the authors were unable to distinguish mesangial cells from SMCs/JG cells and neither obtain sufficient numbers of podocytes from diabetic mice for analysis (34).

**Figure 3:**
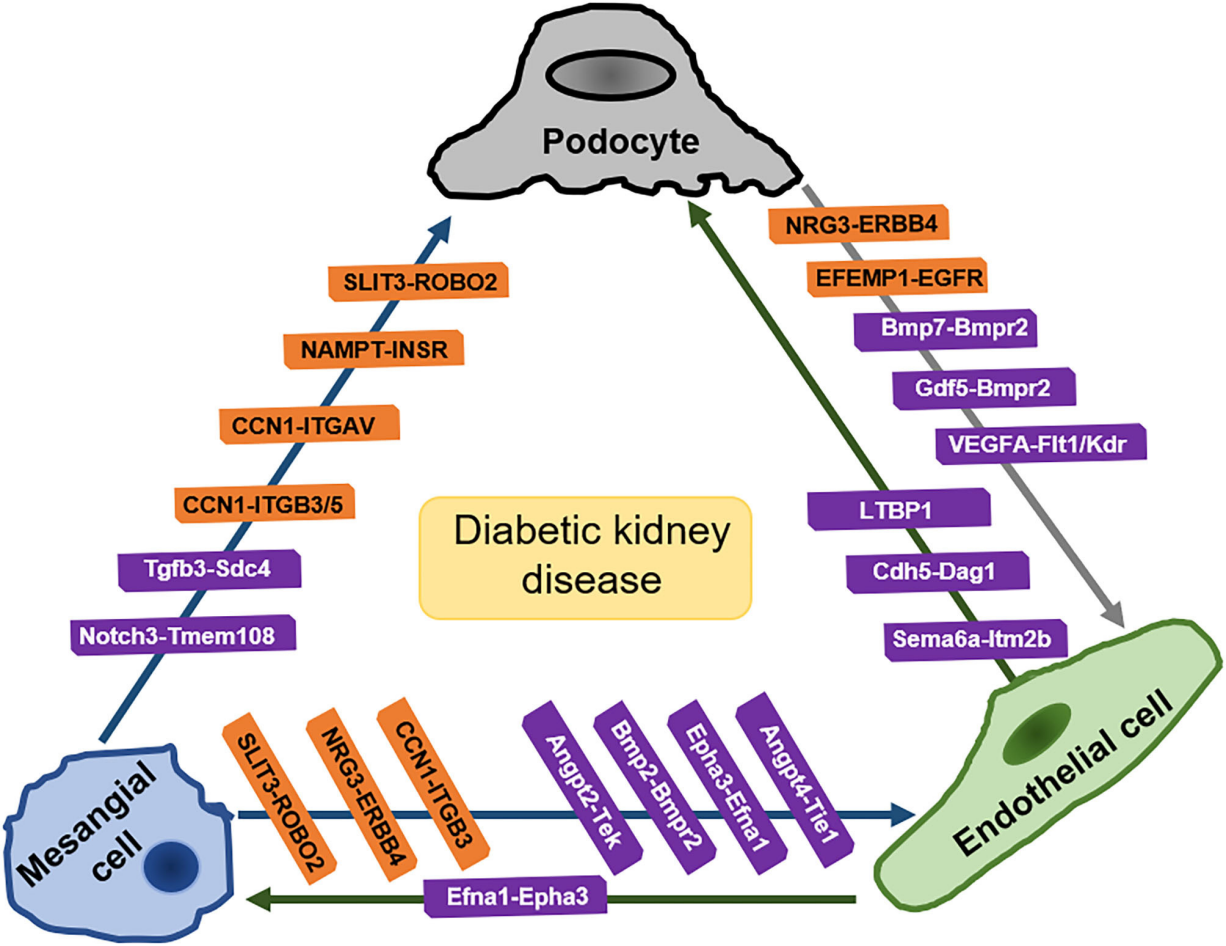
Intraglomerular crosstalk based on ligand-receptor pair analysis of scRNA-seq. Pairs in orange frames come from the analysis of kidney of human with early diabetic nephropathy. Pairs in purple frames come from the analysis of kidney of diabetic mice. SLIT3, slit guidance ligand 3; ROBO2, roundabout guidance receptor 2; NAMPT, Nicotinamide phosphoribosyltransferase; INSR, insulin receptors; CCN1, cellular communication network factor1; ITGB3/5, integrin beta3/5; ITGAV, integrin subunit alpha-V; Tgfb3, Transforming growth factor 3; Sdc4, syndecan 4; Notch3, notch reporter 3; Tmem108, transmembrane protein 108; NRG3, neuregulin-3; ERBB4, Erb-B2 receptor tyrosine kinase 4; Bmp2, bone morphogenetic protein 2; Bmp7, bone morphogenetic protein 7; Bmpr2, bone morphogenetic protein receptor 2; Epha3, erythropoietin-producing hepatocellular carcinoma A3; VEGFA, vascular endothelial growth factor A; Flt1(VEGFR1, vascular endothelial growth factor receptor 1; Kdr (VEGFR2), vascular endothelial growth factor receptor 2; Angpt, angiopoietin; Tie (Tek): angiopoietin 1 receptor; Efnb2, ephrin B2; Efna1, ephrin A1; LTBP1, latent transforming growth factor (TGF)-beta binding protein-1; Cdh5, cadherin 5; Dag1, dystroglycan 1; Sema6a, semaphorin 6a; Itm2b, Integral membrane protein 2B; Cdf5, cycling dof factors 5; EFEMP1, Epidermal Growth Factor-containing Fibulin-like Extracellular Matrix Protein 1; EGFR, Epidermal Growth Factor Receptor. [Pairs in orange are summarized from Mitu et al. (93) and pairs in purple are summarized from Fu et al. (35)].

Intraglomerular crosstalk based on ligand-receptor pair analysis of scRNA-seq are shown in Figure 3.

## Extraglomerular Crosstalk in DKD

Tubular-glomerular interplay, which includes two well-known components, glomerular-tubular balance and tubuloglomerular feedback (TGF), has been demonstrated to play important roles in physiological renal function as well as in DKD (97). Proteins leak from glomeruli and arrive at tubular regions, then leading to further tubular injury, which is caused by the accumulation of proteinuria-inducing reactive oxygen species and various cytokines (98). Sirtuin 1 (SIRT1), a nuclear deacetylating enzyme, which mediates deacetylation of transcription factors and histone, is found being downregulated in proximal tubules preceding podocyte injury in DKD (99). Claudin-1 is a membrane protein involved in the formation of tight junctions and is normally expressed in parietal epithelial cells, which creates tight junctions that might prevent leakage from Bowman's capsule (100). In DKD, the downregulated proximal tubular SIRT1 decreases SIRT1 level in podocytes, thereby leading to the ectopic expression of claudin-1 in podocytes and causing albuminuria (101). Glomerular hyperfiltration is proposed to be resulted from tubular growth and upregulates sodium-glucose cotransporter 2 (SGLT2), which enhances proximal tubular reabsorption, leading a reduction of sodium chloride (NaCl) delivery to the macula densa, therefore increasing GFR *via* TGF response (SGLT2-NaCl pathway) (99–101).

Recently Hasegawa group also reported SGLT2 was elevated during early stages of DKD, which could upregulate intracellular glucose levels in proximal tubules and subsequently decrease SIRT1 expression whereas SGLT2 inhibitors preserved SIRT1 expression (102). SGLT2 inhibitors, suggested Hasegawa, might maintain the proximal tubule-podocyte communication. Other tubular-glomerular communications include a group of exosomes enriched with microRNA (miR) mediating podocyte or proximal tubular cell damage (103).

The communications between macrophages and kidney cells rely much on extracellular vesicles (EV). It was reported that miR-21-5p in macrophage-derived EVs regulated pyroptosis-mediated podocyte injury by A20 in DKD (104). It was also suggested that exosomal miR-19b-3p mediated the communication between injured tubular cells and macrophages, leading to M1 macrophage activation (105). Exosomes from high glucose-treated macrophages were implied to activate GMCs *via* TGF-β1/Smad3 pathway (106).

### Findings Based on scRNA-Seq

A scRNA-seq performed on whole kidney cells from healthy mice revealed specific cell types responding to specific kidney related disorders (30). CKD related genes are strongly enriched in proximal tubules. The researchers identified a transitional cell type between principle cell (PC) and intercalated cell (IC) in collecting duct. Notch regulates the cellular identity of neighboring cells by the expression of either Notch ligands or Notch receptors. Genes encoding Notch ligands were highly expressed in ICs while Notch2 receptor and its transcriptional target Hes1 were shown in PCs with high expression level, suggesting that PCs are the Notch signal-receiving cells in the collecting duct. A higher ratio of PCs to ICs in human diabetic kidney biopsy with increased Notch signaling and HES1 expression suggested a shift toward PCs, which is likely the cause of metabolic acidosis in mouse models and patients with CKD (30). A human kidney scRNA-seq research identified NAMPT expressed in mesangial cells, which regulates insulin secretion in pancreatic β-cells, while uncovered a decreased expression of insulin receptors in diabetic podocytes (95). Interestingly, a single-cell transcriptome profiling performed on BTBR ob/ob mice, which do not develop hypertension, showed those animals had no major changes in endothelial cell gene expression while surprisingly gave the vascular disease stereotype of diabetes (34). The researchers suggested that rather hypertension not diabetes induce transcriptional changes in endothelial cells given the prevalence of hypertension in patients with diabetes (34). They thought hypertension might be more important in injuring endothelial cells. However, there remains confusion since findings to date about kidney crosstalk much developed from researches set under the diabetic milieu. The extraglomerular crosstalks are summarized in Table 2.

**Table 2:** Summary of the mediators for extraglomerular crosstalk in DKD.

**Crosstalk**	**Ligand/Receptor**	**Extracellular vesicles**	**Signal pathway**	**Pathological role in DKD**	**Reference**
Podocyte-Tubular epithelial cell		miR-6538, miR-3474, miR-1981-3p, miR-7224-3p. Let-7f-2-3p		Upregulation of Let-7f-2-3p and downregulation of miR-1981-3p, miR-3474, miR-7224-3p and miR-6538 were detected by RT-qPCR in DKD. These EVs from podocyte may travel through the urinary tract and involved in the extrinsic apoptotic signaling pathway of TECs.	(88)
		miR-221		Podocyte-derived EVs in diabetes acted as key mediators of proximal tubule cell injury and the miR-221 in EVs mediated the cells damage through Wnt/β-catenin signaling.	(103)
Tubular epithelial cell-Podocyte			Sirt1	Sirt1 in tubular epithelial cell protects against albuminuria in diabetes by maintaining NMN concentrations around glomeruli, thus influencing podocyte function.	(102)
			SGLT2-NaCl	Glomerular hyperfiltration is proposed to be resulted from tubular growth and upregulates sodium-glucose cotransporter 2 (SGLT2), which enhances proximal tubular reabsorption, leading a reduction of sodium chloride (NaCl) delivery to the macula densa, therefore increasing GFR *via* TGF response.	(99–101)
Macrophage-Podocyte		miR-21-5p		EVs miR-21-5p secreted from macrophage through inhibition of A20 elevate the inflammasome NLRP3, caspases-1 and IL-1β related to pyroptosis, and augment the production of ROS, thereby causing podocyte injury.	(104)
Tubular epithelial cell- Macrophage		miR-19b-3p		Exosomes enriched with miR-19b-3p mediated the communication between injured TECs and macrophages, leading to M1 macrophage activation and tubulointerstitial inflammation though SOCS-1 pathway.	(105)
Intercalated cell-Principle cell	Notch2-Hes1			Genes encoding Notch ligands were highly expressed in ICs while Notch2 receptor and its transcriptional target Hes1 were shown in PCs with high expression level, suggesting that PCs are the Notch signal-receiving cells in the collecting duct.	(3)

*DKD, diabetic kidney disease; TEC, Tubular epithelial cell; RT-qPCR, real-time polymerase chain reaction; EVs, Extracellular vesicles; IL-1β, Interleukin 1β; ROS, Reactive oxygen species; NLRP3, NACHT, LRR, and PYD domains-containing protein 3; SOCS-1, Suppressor of cytokine signaling-1; Sirt1, Silencing information regulator 2 related enzyme 1; NMN, nicotinamide mononucleotide; SGLT2, sodium-glucose cotransporter 2; NaCl, sodium chloride; Hes1, Hairy and enhancer of split homolog-1; PCs, principle cells; ICs, principle cell*.

## Discussion

scRNA-seq is a powerful tool providing unprecedented insight into cell transcriptome, including deciphering cell-to-cell communication in diseases such as DKD. With the aid of scRNA-seq, some new and complicated cellular interactive in DKD have been revealed as well as some new cell subpopulations have been identified in kidney, which imply some key regulators and therapeutic targets for DKD. Though the technology has achieved great advances, the researchers have to face several challenges during seRNA-seq. The cell isolation protocol needs to be optimized to be more efficient when balancing between cell dissociative efficacy and viability since kidney has relatively dense matrix and some of kidney cells under abnormal conditions are susceptible to loss. The big discrepancy on kidney cell numbers and gene expressions gained in different kidney seRNA-seq researches by now is attributed much to their different dissociation protocols. The types of kidney cell identified in scRNA-seq researches primarily depend on the available cell markers, which may not be specific enough or even yet be revealed. Moreover, the huge volume of complex data generated by seRNA-seq needs appropriate analytical and statistical methods and the interpretation of raw data is determined by the choices of computational tools and databases. Of the most importance, the findings drawn from scRNA-seq need to be validated by subsequent experimental tests.

More researches of scRNA-seq coupled with multiomic approaches are expected in future to gain closer access to profound pathogenesis of DKD and contribute to develop new therapeutic strategies.

## Author Contributions

All literatures were reviewed by FY and CD. Materials were collected by YR, GL, YY, and ZY. The final manuscript was drafted by CD and FY, reviewed by FY and approved by all authors.

## Conflict of Interest

The authors declare that the research was conducted in the absence of any commercial or financial relationships that could be construed as a potential conflict of interest.

## Publisher's Note

All claims expressed in this article are solely those of the authors and do not necessarily represent those of their affiliated organizations, or those of the publisher, the editors and the reviewers. Any product that may be evaluated in this article, or claim that may be made by its manufacturer, is not guaranteed or endorsed by the publisher.
